# A simplified amoxicillin regimen with dose frequency based on post-natal age in neonates with confirmed or suspected infection

**DOI:** 10.1128/aac.01491-24

**Published:** 2025-03-04

**Authors:** Mispah Mukap, Corin Sprod, Okhee Yoo, Nakapi Tefuarani, John Vince, Moses Laman, Madhu Page-Sharp, Brioni R. Moore, Kevin T. Batty, Timothy M. E. Davis, Sam Salman, Laurens Manning

**Affiliations:** 1 School of Medicine, University of Papua New Guinea 119068 , Port Moresby, Papua New Guinea; 2 Papua New Guinea Institute of Medical Research 116896 , Goroka, Papua New Guinea; 3 Pharmacy, School of Allied Health, University of Western Australia, Nedlands, Australia; 4 Curtin Medical School, Curtin University 614117 , Bentley, Western Australia, Australia; 5 Medical School, University of Western Australia, Harry Perkins Research Institute, Fiona Stanley Hospital 418838 , Murdoch, Western Australia, Australia; 6 Department of Medicine, Fremantle Hospital, Fremantle, Western Australia, Australia; 7 Medical School, University of Western Australia, Fremantle Hospital, Fremantle, Western Australia, Australia; University Children's Hospital Münster, Münster, Germany

**Keywords:** amoxicillin, neonates, infection, pharmacokinetic, sepsis, ampicillin

## Abstract

Amoxicillin plus gentamicin is the recommended first-line empiric therapy for neonates with infection. Guidelines vary widely in dose (mg/kg), dose frequency, and adjustments according to post-menstrual age (PMA) and post-natal age (PNA). We aimed to develop a population pharmacokinetic (PK) model for amoxicillin in neonates with clinical evidence of sepsis and design optimal dosing regimens. One hundred seventy-seven neonates receiving intravenous amoxicillin for infection were enrolled in a prospective, observational PK study in Papua New Guinea (PNG). The probability of PK-pharmacodynamic target attainment (PK-PD PTA) was determined based on minimum inhibitory concentrations (MIC) and the proportion of time concentrations that remained above these values (%T > MIC). Neonates with concentrations > 140 mg/L were considered to be at increased risk of amoxicillin neurotoxicity. A population PK model was developed. Simulations tested existing guidelines and proposed simplified regimens. The median PMA and PNA were 38 (37–40) weeks and 0 (0–2) days, respectively. From simulations, existing regimens with 50 or 100 mg/kg doses were associated with higher potential neurotoxic concentrations (24.9% and 84.5%, respectively). With the existing 30 mg/kg PNG regimen, neonates receiving twice-daily dosing between 3 and 7 days were systematically underdosed. A proposed 30 mg/kg regimen, with twice-daily dosing for the first 2 days PNA and three times daily from day 3, provides an optimal balance between the probability of PK-PD target attainment while minimizing toxicity. For fixed volume dosing, using 52 mg (0.25 mL of 250 mg in 1.2 mL) for those <3 kg and 104 mg (0.5 mL) for those ≥3 kg is proposed.

## INTRODUCTION

Neonatal sepsis remains a significant cause of infant morbidity and mortality, particularly in resource-limited settings (1). Early and effective antimicrobial therapy is an essential part of management. Amoxicillin, a broad-spectrum penicillin antibiotic, is often used in combination with gentamicin due to its safety profile, proven efficacy, and low cost. However, selecting the appropriate dosing regimen in neonates is challenging, resulting in highly variable dosing (2). There is a lack of pharmacokinetic/pharmacodynamic (PK/PD) data for amoxicillin (3) and other older antibiotics (4), and studies in neonates and young children are difficult because repeated blood sampling could result in adverse hemodynamic effects (5). Additionally, the results from adult studies in developed countries may not be generalizable to younger age groups in resource-poor settings, due to differences in patient characteristics, effect on drug disposition and metabolism (6, 7), clinical spectrum of disease, and characteristics of the bacterial pathogens (8).

Considering this, there is a growing need for tailored neonatal amoxicillin dosing regimens that account for weight, post-menstrual age (PMA), and post-natal age (PNA). Of these factors, a newborn weight and PNA can be accurately measured, whereas in countries such as Papua New Guinea (PNG), where ultrasound is not routinely available and women present late in pregnancy, the use of PMA is an unreliable tool to predict pre-term delivery (9). Recently evaluated dosage regimens focus primarily on dose and dose frequency that ensures adequate antimicrobial coverage, but there is a balance between achieving therapeutic drug concentrations and avoiding potential toxicity. In the absence of validated individualized regimens, simplified dosing strategies based on age or weight bands are often provided at the health-center level and in national and international guidelines (3), but these may not always ensure therapeutic efficacy and avoid toxicity in neonates with sepsis.

Amoxicillin is used empirically to treat group B streptococci (*Streptococcus agalactiae*), *Listeria monocytogenes,* and enterococci in cases of neonatal sepsis. These organisms have clinical breakpoints or minimum inhibitory concentrations (MICs) of 0.25, 1, and 4 mg/L, respectively (10). The surrogate measure for efficacy for all beta-lactam antibiotics, including amoxicillin, is the time that the free concentrations are above these breakpoints as a percentage of the dosing interval (fT% > MIC). Different authors have recommended fT% > MIC thresholds of 30%, 40%, 50%, 70%, and 100%, or 400% in critical illness as pharmacokinetic-pharmacodynamic (PK-PD) targets for efficacy (11–14).

Amoxicillin has traditionally been considered to have a wide therapeutic index. However, like other beta-lactam antibiotics in critical care settings (15), amoxicillin is now recognized to be associated with neurotoxicity when peak concentrations at steady state exceed 140 mg/L (10, 16). The mechanism for beta-lactam neurotoxicity is thought to be due to the concentration-dependent inhibition of the gamma-aminobutyric acid (GABA) receptor complex (15). The impact of these concentrations on the clinical course and long-term outcomes of neonates with sepsis is unknown, but possible toxicity thresholds should be consideration when developing optimized dosing regimens (10).

Published amoxicillin dosing regimens vary according to body weight from 25 to 100 mg/kg (17, 18). Dose frequency also varies from twice (BD) to four times (QID) daily, usually depending on PNA, with a > 7-day PNA being the most common threshold for higher dose frequency but also occasionally according to PMA (17). As an example, the World Health Organization (WHO) recommends 50 mg/kg BD for those younger than 7 days and three times per day (TDS) for those older than 7 days (19). By contrast, the current pediatric guidelines in PNG recommend 30 mg/kg BD and QID with a PNA of 7 days as the cutpoint for increased dose frequency (20).

To address the lack of high-quality pharmacokinetic (PK) data, particularly from developing countries, we aimed to develop a robust population PK model for amoxicillin in neonates with clinical evidence of confirmed or suspected sepsis and use model-based simulations to design optimal dosing regimens for PNG and comparable settings.

## MATERIALS AND METHODS

### Patients and sample collection

Babies aged ≤2 months presenting to the neonatal unit at PMGH with bacteriologically confirmed or clinically suspected neonatal sepsis were eligible for recruitment. Written informed consent was obtained from the parents or guardian. A baseline assessment was performed, including PNA and PMA, body weight, length, and abdominal circumference. An intravenous (IV) cannula was inserted and 1–5 mL of blood was taken for culture, biochemistry, full blood count, and baseline drug assay. A mixed capillary blood heel-prick sample was taken for the measurement of blood glucose and lactate using point-of-care tests. Per hospital protocols, antibiotics were started without delay after the collection of blood cultures.

Regular monitoring of vital signs and physical examination findings were documented in the standard hospital admission record, including axillary temperature, heart rate, oxygen saturation, respiratory rate, urine output, capillary refill time, abdominal circumference, muscular tone, fontanelle bulging, rash, diarrhea, vomiting, indrawing and subcostal recessions, neck stiffness, lethargy, feeding patterns, and seizure activity. Oxygen therapy, fluid resuscitation, and blood transfusion were given in accordance with national guidelines (20).

Confirmed sepsis was defined as at least one positive bacterial culture from a sterile site, together with at least one of the clinical features and one laboratory feature (see the supplemental material) (21, 22).

Culture-negative sepsis was defined as the presence of at least two clinical and one laboratory parameter from those listed above, without a positive blood culture. Presumed sepsis was defined as the presence of at least one clinical or one laboratory parameter from the list in the supplemental material. These criteria conform with international definitions of neonatal sepsis (21–23).

Amoxicillin was administered intravenously at a dose of 30 mg/kg as a slow injection over 3 min, in accordance with local prescribing guidelines. Powdered amoxicillin (250 mg) was reconstituted with 1 mL water for injection, resulting in a final volume of 1.2 mL due to displacement volume (0.2 mL) of the amoxicillin powder, and the volume as close to the calculated dose of 30 mg/kg as possible was drawn up. At the time of the study, clinical practice at PMGH was to provide BD dosing for the first 7 days, and TDS dosing thereafter. In other remote PNG centers, standard practice was to reconstitute amoxicillin solution as above and draw up fixed volumes of 0.25 mL (a target planned dose of 62.5 mg) for babies weighing <2.5 kg and 0.5 mL (a target planned dose of 125 mg) for those weighing >2.5 kg with the same dosing frequency. Due to displacement volume, the actual doses for 0.25 mL and 0.5 mL are 52 mg and 104 mg, respectively. Gentamicin was also administered in accordance with the local clinical guidelines.

Mixed capillary blood from heel pricks was collected onto filter paper cards (Whatman 903 Protein Saver Cards, GE Healthcare Pty Ltd, NSW, Australia) at baseline, 0.5, 1, 4, 12, 24, and 48 h following the first dose of amoxicillin. The dried blood spot (DBS) cards were air dried at room temperature for 1 h, placed in an airtight foil envelope with a single desiccant sachet, and transported on ice before storage at −80°C.

At 24 h, before the third or the fourth dose was due and when a “trough” concentration was expected, a 1.5 mL sample of venous blood was collected with prior removal of up to 3 mL blood to eliminate dead space effects. The total volume of blood drawn from each baby was in accordance with international recommended limits for pediatric research and approved by the Human Research Ethics Committee (HREC) (5). After centrifugation, plasma and red cell pellets were separated and kept on ice before storage at −80°C.

We developed liquid chromatography-mass spectroscopy (LC-MS-MS) methods to measure amoxicillin and gentamicin simultaneously from plasma and DBS. Analytical assay details are provided elsewhere, but the limits of detection (LOD) and quantification (LOQ) for amoxicillin were 0.02 mg/L and 0.05 mg/L for plasma and DBS amoxicillin assays (24).

Due to low protein binding, we assumed that plasma amoxicillin concentrations reflected free concentrations (25). For assessments of the probability of target attainment, we chose the time that free concentrations of amoxicillin exceeded the MIC (fT >MIC) across a range from 10% to 100% of the time over a 24 h period as the PK-PD surrogate for efficacy. We chose the breakpoint MICs of 0.25, 1, and 4 mg/L to best reflect breakpoints for representative neonatal pathogens (26).

### Pharmacokinetic modeling

The analysis was carried out using NONMEM (v 7.5.1, ICON Development Solutions, Ellicott City, MD, USA), leveraging a Gfortran 4.6.0 compiler (for full details, see the supplemental material).

### Model evaluation

A bootstrap procedure was executed using Perl speaks NONMEM (PSN) with 1,000 samples. The parameters derived from this analysis were summarized as median and 2.5^th^ and 97.5^th^ percentiles (representing a 95% empirical confidence interval) to aid in assessing the final model parameter estimates. In addition, prediction-corrected visual predictive checks (pcVPCs) were carried out with 1,000 data sets, simulated from the final models. These simulations were stratified according to weight, PMA, and PNA. The observed 10th, 50th, and 90th percentiles were graphed alongside their corresponding simulated 95% confidence intervals to evaluate the predictive capability of the model.

### Simulations

Once the final population pharmacokinetic model was defined, Monte Carlo simulations were conducted to explore the impacts of current and potential alternative dosing regimens. Using weight-for-age statistical data (27) for preterm infants, simulations were performed on 1,000 male and 1,000 female infants each for PNA less than 3 days, PNA 3–7 days, and over 7 days, and for each week of PMA between 33 and 41 weeks. This simulation structure was applied for BD, TDS, and QID dosing. The resulting data set was analyzed using Power BI (Version: 2.114.864.0, Microsoft). The DBS concentrations were used for model building. After the DBS model was completed, a correction ratio for plasma samples was incorporated into the model, as plasma concentrations were obtained as a single point and were not intended for the final PK model construction. Using this predicted plasma-to-DBS ratio, the simulated DBS concentrations were then adjusted to free plasma concentrations by multiplication. The current dosage regimen for PNG was stratified at PNA 7 days, and the alternative regimens considered include dosage adjustments at PNA 3 days with the aim of achieving more precise target attainment, as well as a single BD dosing regimen which followed the existing WHO guidelines for a more convenient and practical regimen. The investigated dosing strategies are outlined below:

1. A modified WHO dose of 50 mg/kg BD until day 7 then TDS (19).
2. The existing PNG dosing of 30 mg/kg BD until day 7, then QID (20).
3. A “ meningitis” dose of 100 mg/kg BD until day 7, then TDS (17, 18).
4. A proposed new dose regimen of an actual dose of 30 mg/kg BD for the first 48 h (“Two for two”), followed by TDS dosing from day 3 (“Three from three”).

We explored scenario 4 to determine the effect of fixed dosing across different weight bands by examining interactive changes in PTA using Power BI. This included doses of 0.25 mL (62.5 mg) and 0.5 mL (125 mg) of reconstituted amoxicillin for those weighing <2.5 kg or 2.5–4 kg in accordance with the existing weight thresholds on the PNG standard treatment guidelines. We also propose a new weight threshold of <3 kg or 3–4 kg for these fixed doses. The choice of weight threshold was based on evaluation of the simulated data and reflects a value that is easily measured and remembered. We then repeated the scenario 4 analyses to account for possible dosing inaccuracies if the injection resulted in a final volume of 1.2 mL rather than 1 mL (to account for the displacement volume of amoxicillin powder), from which 0.25 mL (52 mg) and 0.5 mL (104 mg) were administered.

## RESULTS

### Participant characteristics

A total of 177 neonates were recruited. The median (interquartile range [IQR]) weight, PNA, recorded gestation at birth were 2.8 (2.1–3.3) kg, 0 (0–2) days, and 38 (37-40) weeks, respectively (Table 1). Hematocrits ranged from 0.06 to 0.74 (median: 0.49 [0.43–0.55]). The median lactate was 3 (2.2–4.4) mmol/L. Sixteen (9.0%) had culture-confirmed sepsis with a significant organism isolated from blood culture. This included coagulase-negative staphylococci (*n* = 10; 58%), *Klebsiella pneumoniae* (*n* = 3, 17.6%), *Staphylococcus aureus* (*n* = 1, 5.8%), and *Pseudomonas aeruginosa* (*n* = 1, 5.8%). The MIC value for common antibiotics for these organisms was not available.

**TABLE 1:** Baseline characteristics of neonates (*n* = 177)

	Median (IQR [range]) or number (proportion)
Sex (%) male	57^*a*^
GA (weeks)	38 (37–40 [28 – 42])^*b*^
PNA (days)	0 (0–2 [0–48])
PMA (days)	273 (260–280 [196 – 328])^*c*^
Weight (kg)	2.8 (2.1–3.3 [0.9–5.5])^*d*^
Height (cm)	46 (42–48 [6 – 59])^*e*^
Dose amoxicillin (mg)	80 (60–96 [15 – 96])^*f*^
Weight-adjusted dose amoxicillin (mg/kg)	30 (28.1–30 [9.4–59])

^*a*^ 12 unknown sex.

^*b*^ GA missing for 15 participants.

^*c*^ For PMA, missing GA replaced with median GA.

^*d*^ Weight missing for 11 participants.

^*e*^ Height missing for 11 participants.

^*f*^ Amox dose missing for 4 participants.

Of the remaining, 108 (61.0%) and 53 (30.0%) had sepsis confirmed by clinical findings or at least one indicator of sepsis, respectively. Twelve (6.8%) children died.

Of 1216 DBS samples available, 71 (5.8%) were excluded because the drug was either detectable at time zero (28) or “peak” concentrations were observed at times predicted to be trough concentrations (42). A total of 135 paired DBS and venous samples were analyzed, with Spearman’s rho 0.77, indicating a high positive correlation across all data points (Fig. S1l) (29). Bland-Altman analysis uncovered heteroscedasticity with the between-sample difference at higher concentrations exceeding that at lower concentrations. The log-transformed concentration of 134 data points demonstrated a random distribution with a bias of 0.73 in the log concentration difference (95% CI: −2.15 to 0.68) (Fig. S2). Consequently, the amoxicillin concentration ratios between DBS and plasma were 0.48 (95% CI: 0.11–0.97), closely related to the average haematocrit value of 0.49.

### Pharmacokinetic modeling

Therefore, a total of 1,145 DBS and 134 plasma concentrations contributed to the final model. A two-compartment model with first-order elimination was sufficient, showing no notable enhancements in Objective function value (OFV) or diagnostic plots with the addition of another compartment. It was possible to estimate both IIV and inter-occasion variability (IOV) for total clearance (CL) and central volume of distribution (V_c_). When assessing the maturation of clearance as a function of PMA, an exponential model outperformed other tested models. We estimated an 8.5% increase per week in amoxicillin clearance based on PMA. Moreover, a PNA for babies > 3 days old further increased clearance by 57%. No other significant covariate relationships were discovered. The final model parameter estimates are presented in Table 2. Basic goodness-of-fit plots and visual predictive checks (VPC), stratified by weight, PNA, and PMA, are presented in Fig. S3 to S7. The model showed similar predictive performance in stratified pcVPCs. The model slightly overestimated both the 2.5^th^ and 97.5^th^ percentiles in the absorption phase and slightly underestimated the 2.5^th^ percentile in the elimination phase. However, the overall median value was adequately predicted without any bias.

**TABLE 2:** Final population pharmacokinetic estimates and bootstrap results amoxicillin from dried blood spot (DBS) for neonates with sepsis^*a*^

Parameter	Mean	RSE%	Bootstrap median [95% CI]
Objective function value	−311.262		−343.57 [−512.232 to 158.115]
Structural model parameters			
CL, lh^−1^2.8 kg^−1^ = θ_1_ *F_MAT_*CLCov			
θ_1_	0.856	4.8	0.851 [0.780–0.944]
V_C_, l 2.8 kg^−1^	4.11	4.2	4.09 [3.12–4.43]
Q, lh^−1^2.8 kg^−1^	0.406	12.3	0.410 [0.316–0.521]
V_P_, l 2.8 kg^−1^	1.35	8.8	1.35 [1.14–1.64]
Ratio in plasma to in DBS	0.783	**7**	0.788 [0.664–0.895]
Covariate relationships (%)			
F_MAT_ = EXP(θ_exp, PMA_ *(PMA-39))			
θ_exp, PMA_	0.0816	17	0.0817 [0.0543–0.1108]
Cov			
PNA <= 2, CLCOV = 1			
PNA > 2,CLCOV=(1+θ_Dichotomous,PNA_)			
θ_Dichotomous, PNA_	0.57	27.7	0.254 [0.192–0.379]
Interindividual variability [shrinkage%]			
CL	49.5 [3]	9.8	48.7 [39.7–58.4]
V_C_	42.2 [21]	13.8	42.0 [29.5–52.9]
Ratio	47.7 [50]	18	48.2 [0.52–61.2]
Interoccasional variability			
CL	31.8 [27]	8	31.7 [27.0–36.6]
V_C_	34.6 [71]		34.1 [20.1–46.8]
Residual errors			
AddLn_DBS_	18.2 [40]	1	18 [16–21]
Prop_Plasma_	58.4 [35]	13	55 [29–84]

^*a*^ CI, confidence interval; CL, total clearance; F_MAT_, maturation of clearance; V_C_, central volume of distribution; Q, intercompartmental transfer; V_P_, peripheral volume of distribution; PMA, postmenstrual age in weeks; PNA, postnatal age in days; AddLnDBS, additive error in log-transformed DBS; AddLnPlasma, additive error in log-transformed plasma; % RSE, relative standard error [%RSE = 100 × (standard error/parameter estimate)]; inter-individual variability and residual error are presented as 100% ×√(variability) estimate.

**Fig 1:**
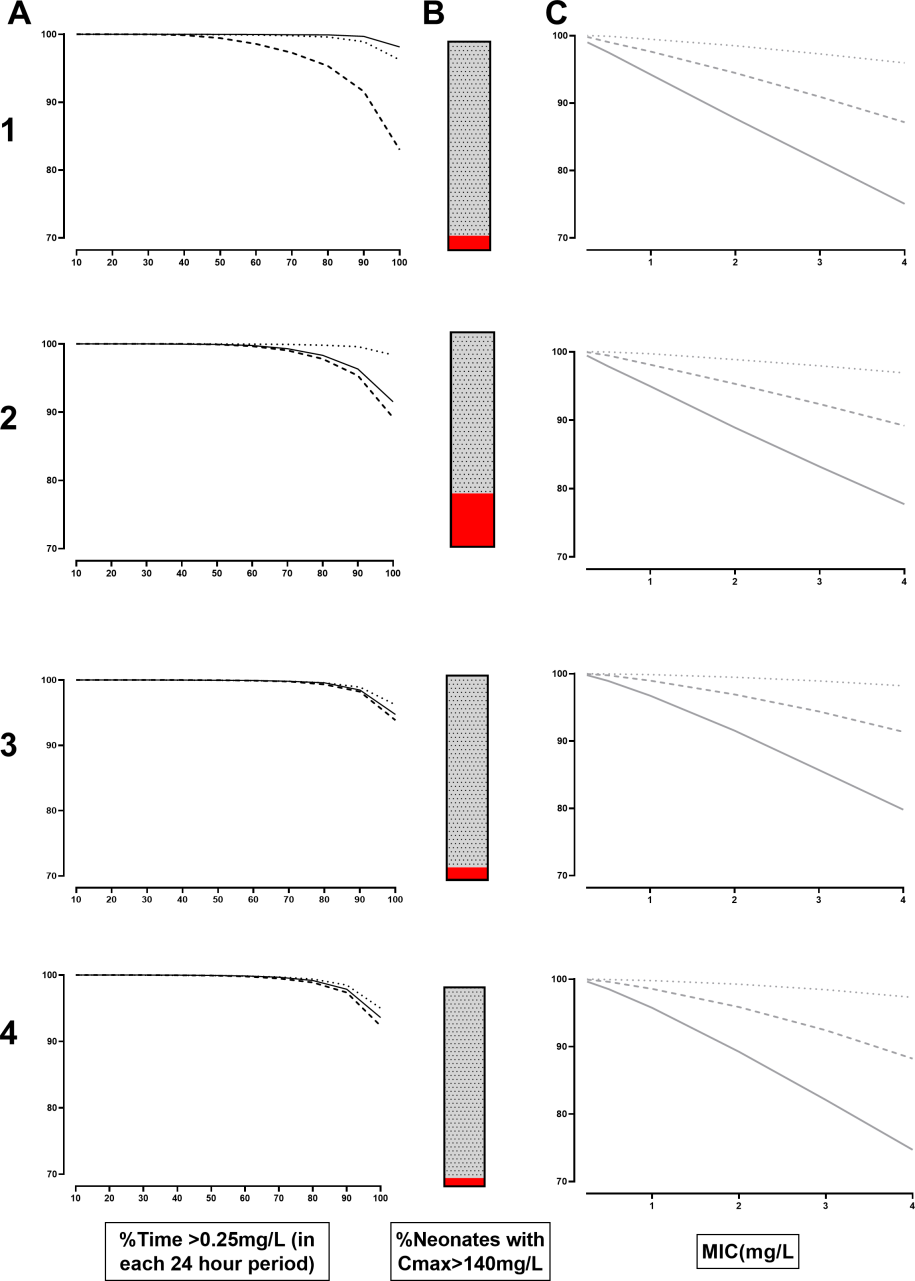
(A1-A4) Probability of target attainment (PTA) plotted against the fraction of time with a minimum inhibitory concentration (MIC) greater than 0.25 mg/L (%ft >0.25) for amoxicillin. Neonates with post-natal age (PNA) of 0–2 days (solid line), 3–7 days (dashed line), and >7 days (dotted line) are depicted. Different dosing regimens from top to bottom are shown including existing Papua New Guinea (30 mg/kg doses, twice daily [BD] for neonates with PNA 0–7 days, and four times daily [QID] for those with PNA >7 days; Row 1) (20), World Health Organization (50 mg/kg BD for neonates aged 0–7 days, and TDS with PNA >7 days; Row 2 [19]), a proposed new PNG dose regimen (30 mg/kg BD for PNA 0–2 days and TDS from PNA of 3 days; Row 3) and a fixed volume according to weight, accounting for dosing inaccuracies (0.25 mL of 250 mg/1.2 mL for those <3 kg, and 0.5 mL >3 kg; Row 4). (B1-B4) Percentage of neonates with concentrations > 140 mg/L, a surrogate for neurotoxicity according to the dosing regimens in Rows 1–4, above. (C1-C4) PTA plotted against MIC according to dosing regiments in Rows 1–4, above. Proportion of neonates with 30% (dotted line), 50% (dashed line), and 70% (solid line) time >MIC is shown.

Simulations for the existing PNG dosing and WHO recommendations are shown (Fig. 1; A1 and A2, respectively). When anchored to PTA for group B streptococci (breakpoint: 0.25 mg/L), the PNG dosing strategy resulted in a drop off in median efficacy for children between 3 and 7 days. The increased dose recommended by WHO pushed the curve to the right, but with a concurrent increase in the proportion at risk of neurotoxicity (24.9% vs 6.9%; Fig. 1, panels B1 and B2, respectively). Where doses of 100 mg/kg were used as a “meningitis-dose,” the proportion of neonates at risk of neurotoxicity increased to 84.5% (data not shown).

Systematic underdosing for neonates aged 3–7 days with the existing PNG guidelines resulted in 94.% and 75.1% of neonates attaining fT% > 70% for breakpoints of 1 and 4 mg/L, respectively (Fig. 1, panel C1). Simulations of a new proposed dose of 30 mg/kg given BD for the first 48 h and TDS thereafter improved the probability of target attainment (PTA; Fig. 1, panel A3) without increasing the chance of neurotoxicity (6.1%; Fig. 1, panel B3). For neonates aged 3–7 days, this regimen resulted in 95.6% and 77.4% attaining fT% > 70% for breakpoints of 1 and 4 mg/L, respectively (Fig. 1, panel C3).

The PTA for a fixed dose with 2.5 kg as the cutpoint for 0.25 and 0.5 mL dosing and assuming that the final volume of the amoxicillin IV solution was 1 mL are shown for the current regimen (62.5 mg and 125 mg) had high PTAs. However, using this regimen resulted in a higher proportion with neurotoxic concentrations (13.2% and 14.1%, respectively; data not shown).

However, if overage is considered, and the fixed doses are 0.25 or 0.5 mL from a total ampoule volume of 1.2 mL, the total dose is reduced to 52 mg and 104 mg. Furthermore, if the weight band for increased volume is increased from 2.5kg to 3 kg, the proportion with neurotoxic concentrations drops to 4.1% while maintaining high PTA (Fig. 1, panels A4, B4, and C4).

## DISCUSSION

This study has demonstrated that the existing PNG and WHO regimens may systematically underdose neonates aged between 3 and 7 days. Due to the recommended increase in dose frequency from BD initially to either TDS or QID dosing is delayed until a PNA of 7 days. Although this may have a limited effect on therapeutic efficacy for infections with group B streptococci where the target MIC is 0.25 mg/L, the likelihood of attaining the PK-PD target with currently recommended regimens decreases markedly for pathogens with higher MICs such as *L. monocytogenes* or enterococci. Our data show that setting a PNA of 3 days as a “clearance maturation” cut-point improved PTA when the target was 0.25 mg/L in our population group, with a median PMA of 39 weeks. This finding accords with a recent sparse sampling PK study in 187 neonates which demonstrated a high PTA using a target MIC of 1 mg/L for BD dosing for neonates with early onset sepsis (EOS; <3 days PNA) and the need for more frequent dosing for older children (28).

The asymptotic exponential and sigmoid Emax maturation functions are standard approaches for modeling neonatal data (30, 31). However, these functions produced poor precision in clearance estimates within our data set, with relative standard errors exceeding 100%. Complete maturation generally occurs around 1 year after birth, highlighting the importance of studying an age range that encompasses the entire S-curve of the sigmoidal function, extending up to 1 or 2 years of PMA (i.e., 52–104 weeks PMA) to reliably estimate sigmoidal maturation. In our case, the available data range of 28–47 weeks PMA, with a median of 39 weeks, was centered around the inflection point of the sigmoidal maturation function (32). Conversely, the exponential function provided a good fit within the region of most rapid maturation. Therefore, our model is considered plausible within the narrow PMA range of up to 1 week (i.e., PNA up to 7 days).

The optimal amoxicillin dosing regimen represents a trade-off between clinical efficacy in terms of PTA against the most common pathogens and possible neurotoxicity, which may constrain upper dose limits. At the same time, maturation of amoxicillin clearance in early life has implications for choosing the best PMA and PNA for increased dose frequency. Consideration is needed for an adaptable pragmatic approach in a resource-limited setting, where responsibility for medication accuracy and clinical decision-making falls upon community health workers. Our aim was to develop an amoxicillin-dosing regimen tailored for use in PNG, but applicable for other clinical contexts. In this setting, including weight and PNA in the model, rather than PMA, is a pragmatic approach that accounts for the fact that recall of the last menstrual period is unreliable as a tool to predict gestational age (9). This included consideration of fixed volume dosing according to body weight categories for use in remote areas that accounted for possible dose inaccuracies when calculated volumes are drawn up after 1 mL diluent is added to a powdered formulation.

Recently, the concept of a neurotoxicity threshold for amoxicillin is a relatively recent consideration. Data from animal studies (33), pre-term neonates (34), and other settings (16), as well as from the use of other beta-lactam antibiotics in high-risk, adult populations (35, 36), have not informed current amoxicillin-dosing regimens for neonates. Applying a recently published amoxicillin PK model to a number of international guidelines, and consistent with the simulations from the present data, a recent simulation-based study demonstrated unacceptably high rates of neurotoxic concentrations (>80%) with 100 mg/kg dosing. In this study, approximately 10%–25% of neonates dosed with 50 mg/kg attained concentrations > 140 mg/L while lower doses of 10–30 mg/kg were associated with lower proportions of potential neurotoxicity.

Consistent with the present study, these observations strongly suggest that a dose of 30 mg/kg results in infrequent (<5%) neurotoxic concentrations, and doses of 50–100 mg/kg would result in an unacceptably high risk of neurotoxicity. Importantly to note, although the adverse impact of high amoxicillin concentrations on outcomes has not been firmly established, they could worsen the poor neurodevelopmental outcomes observed in neonates treated for possible- and culture-positive sepsis (10, 37).

Direct comparisons of our data with the body of literature for amoxicillin PK in neonatal sepsis are difficult due to marked differences in mg/kg doses, allometric scaling of body mass, PNA, and PMA (25). Nevertheless, our point CL estimate of 0.31 L/h/kg is very similar to a recent study in Chinese infants with a similar PMA, weight, and PNA (38). However, the CL reported for pre-term infants in other comparably large population PK studies is lower than that in our model (39).

Based on these data, we have proposed a new dosing regimen for PNG, which has been based on discussions with the PNG collaborators. Ideally, however, the calculus of the relevant trade-off between efficacy, toxicity, and regimen simplicity needs to be tailored to each setting.

The present study also explored the impact of fixed-volume dosing according to weight-based categories that are provided as a pragmatic alternative to mg/kg dosing in PNG and WHO guidelines (19, 20). A simple shift from a cutoff of 2.5 kg to 3 kg for increasing the dose from 0.25 mL to 0.5 mL and applying a TDS dosing from day 3 maintained PK-PD efficacy parameters and minimized potentially neurotoxic concentrations.

We also accounted for dose inaccuracies that arise from applying fixed volume dosing where the addition of diluent (1 mL) to the powdered 250 mg amoxicillin preparation results in a final volume of 1.2 mL, so that the final concentration is 208 mg/mL. The net result of this is the systematic delivery of lower doses when fixed volumes are used (52 mg vs 62.5 mg for 0.25 mL of a solution containing 250 mg in 1.2 mL and 104 mg vs. 125 mg for 0.5 mL of the same solution). As demonstrated, this results in the lowest observed proportion of infants achieving neurotoxic concentrations while maintaining efficacy.

Our study has some limitations. First, more than three-quarters of babies were term babies presenting with early onset sepsis, within the first 48 h of birth. Only 9.0% had sepsis confirmed with blood cultures, whereas the remainder had clinical signs consistent with probable sepsis. Hence, there may be less generalizability to babies presenting with late-onset sepsis and those with extreme prematurity or culture-confirmed sepsis. Finally, we acknowledge that the proposed regimen should be evaluated in future studies of acceptability (for health workers), clinical efficacy, and toxicity.

A proposed 30 mg/kg regimen with twice-daily dosing for the first 2 days and three times daily from the third day provides the best balance between the probability of PK-PD target attainment while minimizing toxicity and maintaining simplicity. Where fixed-volume dosing according to weight is preferred, we propose a dosing of 52 mg (0.25 mL of 250 mg in 1.2 mL) for those <3 kg and 104 mg (0.5 mL) for those ≥3 kg.

## SUPPLEMENTAL MATERIAL

Supplemental material aac.01491-24-s0001.docx Additional description of sepsis definitions; Figures S1 to S7.

ASM does not own the copyrights to Supplemental Material that may be linked to, or accessed through, an article. The authors have granted ASM a non-exclusive, world-wide license to publish the Supplemental Material files. Please contact the corresponding author directly for reuse.
