# Supplementary material for: A simplified amoxicillin regimen with dose frequency based on post-natal age in neonates with confirmed or suspected infection
Source: Antimicrob Agents Chemother. 2025 Mar 4;69(4):e01491-24. doi: 10.1128/aac.01491-24 (PMC11963543; doi:10.1128/aac.01491-24)
Supplement: Supplemental material — Additional description of sepsis definitions; Figures S1 to S7. [file aac.01491-24-s0001.docx]

**A simplified amoxicillin regimen with dose frequency based on post-natal age in neonates with confirmed or suspected infection**

**Supplementary material**

**1. Inclusion criteria for neonatal sepsis**

Clinical and laboratory features:

i) modified body temperature: hyperthermia ≥38.5°C or hypothermia <36°C or temperature instability, ii) cardiovascular instability: bradycardia <123 bpm or tachycardia >171 bpm, reduced urinary output, mottled skin, or impaired peripheral perfusion, iii) rash, iv) respiratory instability: apnoeic episodes or bradypnoea <25 breaths/min, tachypnoea >51 breaths per min, or SpO_2_ ≤92% on room air, v) feeding intolerance or abdominal distention and vi) non-specific signs: irritability, lethargy, hypotonia or laboratory signs which included peripheral white cell count <4 or >20 × 10^9^/L, platelet count <100 × 10^9^/L, hyperglycaemia >10 mmol/L, hypoglycaemia <2.5 mmol/L, or hyperlactataemia (serum lactate >2 mmol/L) (1, 2).

**2. Pharmacokinetic modelling**

Parameter estimation was performed utilising the first order conditional estimation (FOCE) with interaction method. The process of model selection was guided by the minimum value of the objective function (OFV), conditional weighted residuals (CWRES) plots, and visual diagnostic plots. A *P*-value of <0.01 was set as the significance level for comparing nested models. Residual variability (RV) was estimated as additive error for the log-transformed data. Both inter-individual variability (IIV) and inter-occasion variability (IOV) terms were added to parameters, wherever it was reasonable based on the data.

Initial models were created focusing solely on amoxicillin DBS, exploring variations with one, two, and three compartments, all of which employed first-order elimination. Each dose was considered as an individual event, thus incorporating inter-occasion variability into the clearance and volume of distribution. Allometric scaling was pre-established, taking into account body weight (WT, in kilograms), with set exponents of ¾ for clearance and 1 for volume. Different maturation factors on clearance were examined as functions of the postmenstrual age (PMA).

The following maturation factors were examined:

(i) a linear model:

F_mat_ = 1 + θ_lin_ $\times$ (PMA ­- PMA_median_)

where θ_lin_ is the parameter describing the linear change of clearance with PMA and PMA_median_ is the median of PMA in the population;

(ii) an exponential model:

F_mat_ = $e^{\theta\exp\times(PMA - PMA\mathrm{median} )}$

where θ_exp_ is the parameter defining the exponential change in clearance with PMA;

(iii) an asymptotic exponential model:

F_mat_ = (1– (1 – β_CL_)) $\times$ $e^{-\left( \mathrm{PMA}- PMA\mathrm{median} \right)\times\frac{Ln2}{Tcl}}$

where β_CL_ is the fractional estimate of clearance at the median PMA while T_cl_ is the maturation half-life; and finally

(iv) a sigmoid E_max_ model:

F_mat_ = $\frac{{PMA}^{HILL}}{{PMA}^{HILL} {+ MAT}_{50}^{HILL}}$

where Hill is the Hill coefficient and MAT_50_ is the PMA where 50% maturation occurs.

Once the structural model was established, potential covariates were evaluated through the inspection of plots depicting covariate against individual parameters, along with the evaluation of physiological plausibility. Following this, potential covariates were tested within NONMEM using a stepwise forward (with a significance level of *P* < 0.05) and backward (with a significance level of *P* < 0.01) approach.

Initial model building was conducted using DBS concentrations. Upon completing the DBS model, we concurrently fitted the model to both plasma and DBS data, introducing a correction ratio for plasma samples. This estimated ratio facilitated the conversion from DBS to plasma in the simulations.

**3. Supplementary figures**

**Figure S1**. Linear Relationship Between Plasma and Dried Blood Spot (DBS) Concentrations: Spearman's Rho = 0.77


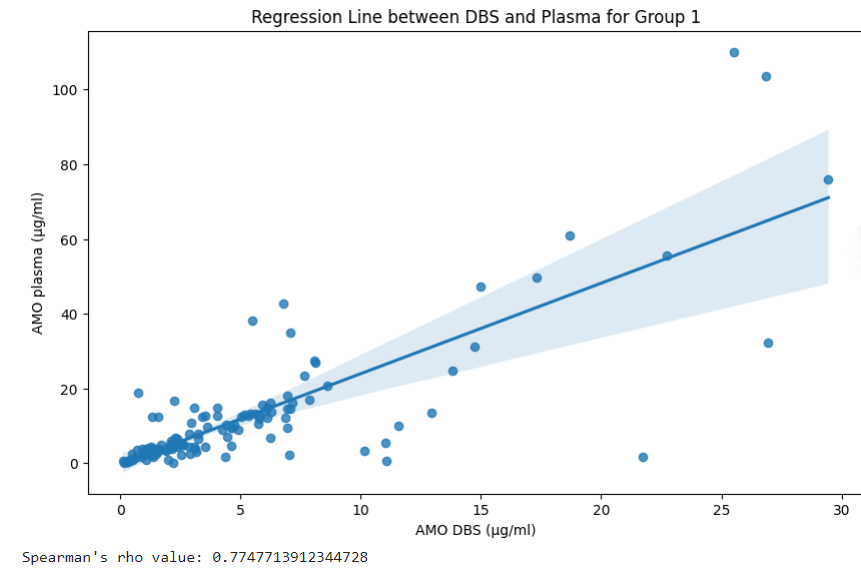


**Figure S2**. The Bland-Altman plot depicts the log transformed amoxicillin concentrations from plasma (log[Plasma]) and dried blood spot (log[DBS]). The dashed line represents the mean difference between log[Plasma] and log[DBS], while the dotted lines define the 95% limits of agreement. Observed values are denoted by blue dots, with log transformations using the natural logarithm (base e).


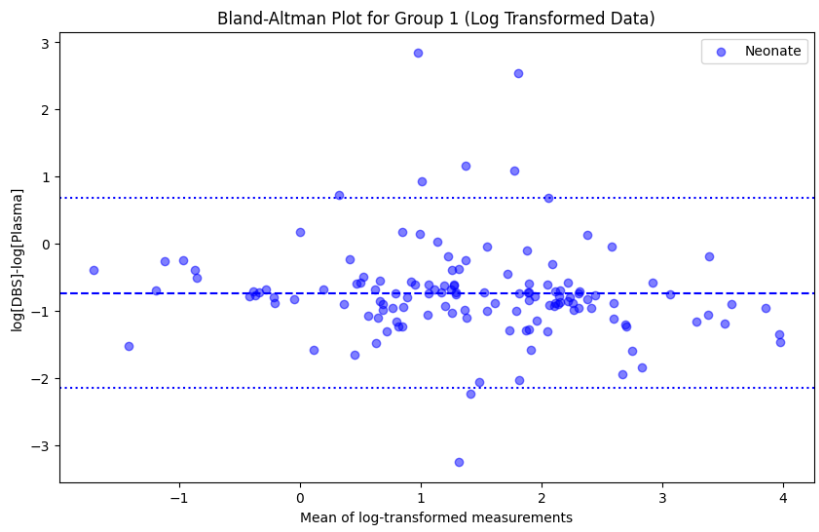


**Figure S3**. Goodness-of-Fit Plots: IV Amoxicillin in PNG Neonates with Sepsis


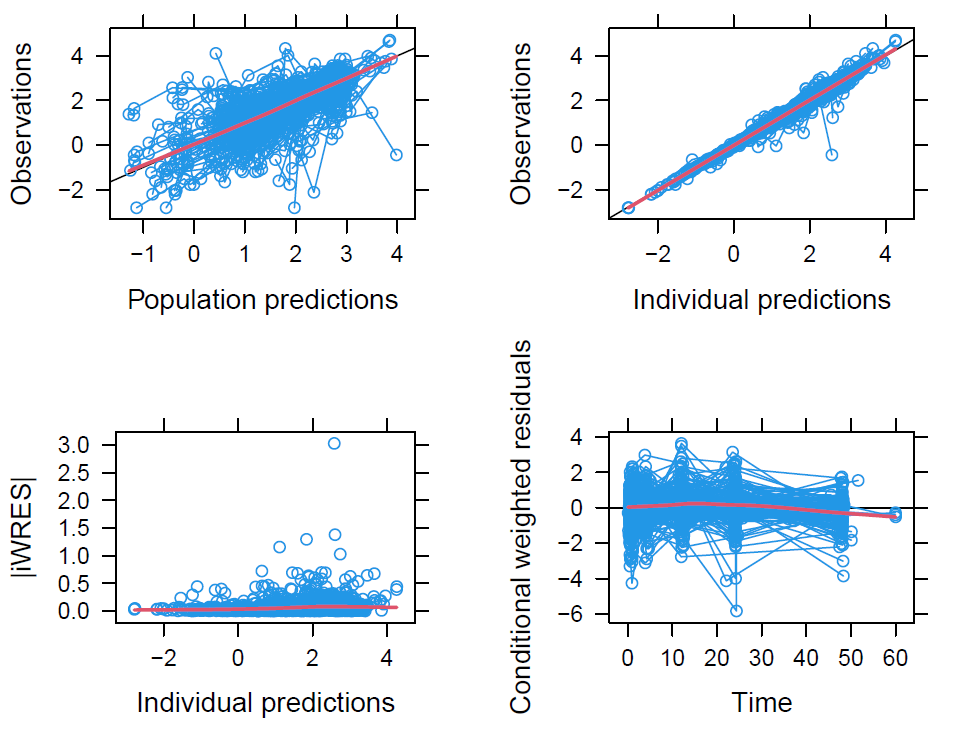


**Figure S4.** Prediction corrected Visual Predictive Check (VPC) for amoxicillin final model. Observed 50th (solid line), 10th and 90th (dotted lines) percentiles within their simulated 95%CI (shaded areas) with overlying data points (○)


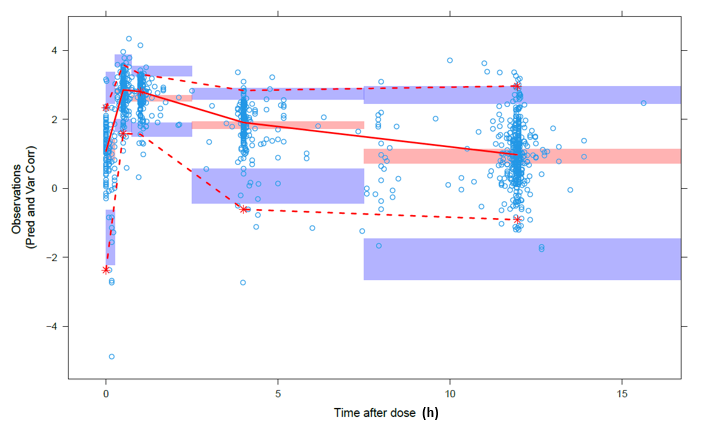


**Figure S5.** Prediction corrected Visual Predictive Check (VPC) for amoxicillin final model Stratified by weight (WT) (kg). Observed 50th (solid line), 10th and 90th (dotted lines) percentiles within their simulated 95%CI (shaded areas) with overlying data points (○)


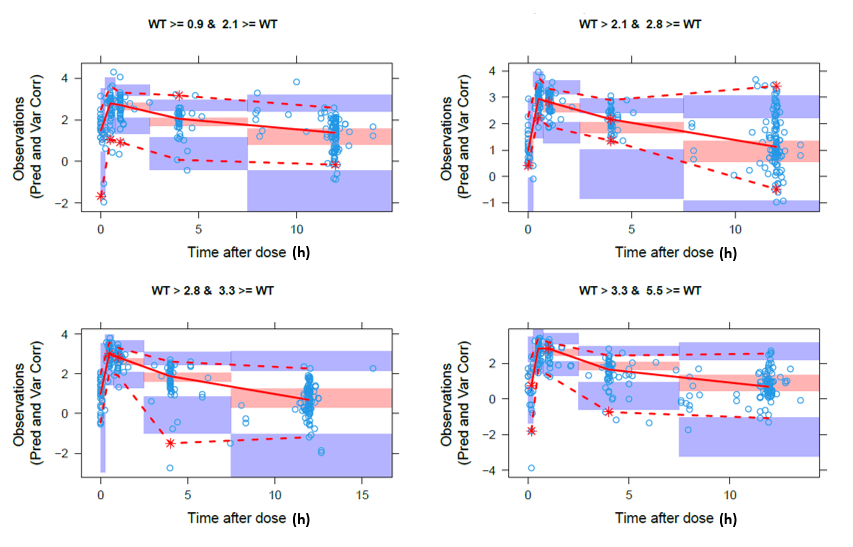


**Figure S6.** Prediction corrected Visual Predictive Check (VPC) for amoxicillin final model stratified by postnatal age (PNA) in days. Observed 50th (solid line), 10th and 90th (dotted lines) percentiles within their simulated 95%CI (shaded areas) with overlying data points (○)


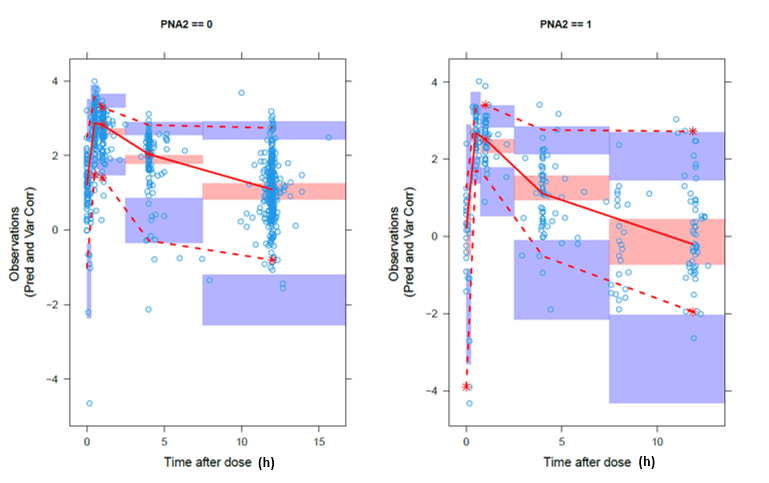


**Figure S7.** Prediction corrected Visual Predictive Check (VPC) for amoxicillin final model Stratified by postmenstrual age (PMA) in days. Observed 50th (solid line), 10th and 90th (dotted lines) percentiles within their simulated 95%CI (shaded areas) with overlying data points (○)


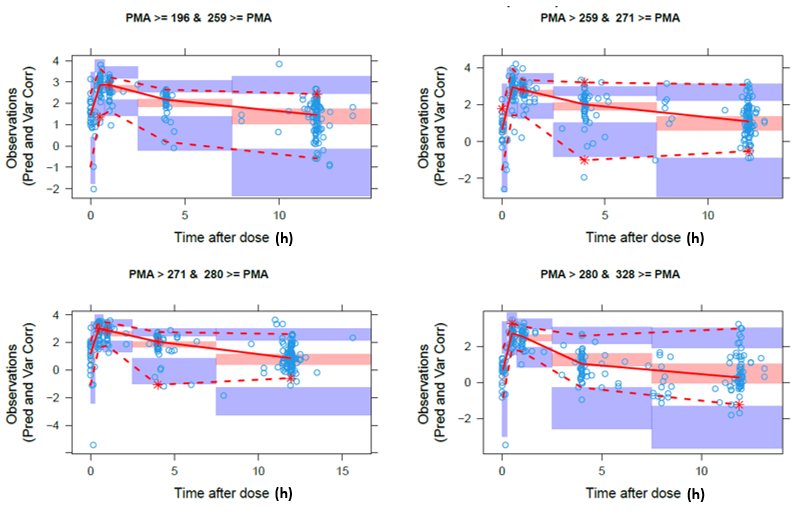


**References**

1. Report on the expert meeting on neonatal and paediatric sepsis in London.: European Medicines Agency; 2010.

2. O'Leary F, Hayen A, Lockie F, Peat J. Defining normal ranges and centiles for heart and respiratory rates in infants and children: a cross-sectional study of patients attending an Australian tertiary hospital paediatric emergency department. Arch Dis Child. 2015;100(8):733-7.

1. Salman S, Hibbert J, Page‐Sharp M, Manning L, Simmer K, Doherty DA, et al. Effects of maturation and size on population pharmacokinetics of pentoxifylline and its metabolites in very preterm infants with suspected late‐onset sepsis or necrotizing enterocolitis: a pilot study incorporating clinical outcomes. British Journal of Clinical Pharmacology. 2019;85(1):147-59.
